# Supplementary figures and images for: Synergy of the microRNA Ratio as a Promising Diagnosis Biomarker for Mucinous Borderline and Malignant Ovarian Tumors
Source: Int J Mol Sci. 2023 Nov 6;24(21):16016. doi: 10.3390/ijms242116016 (PMC10649586; doi:10.3390/ijms242116016)

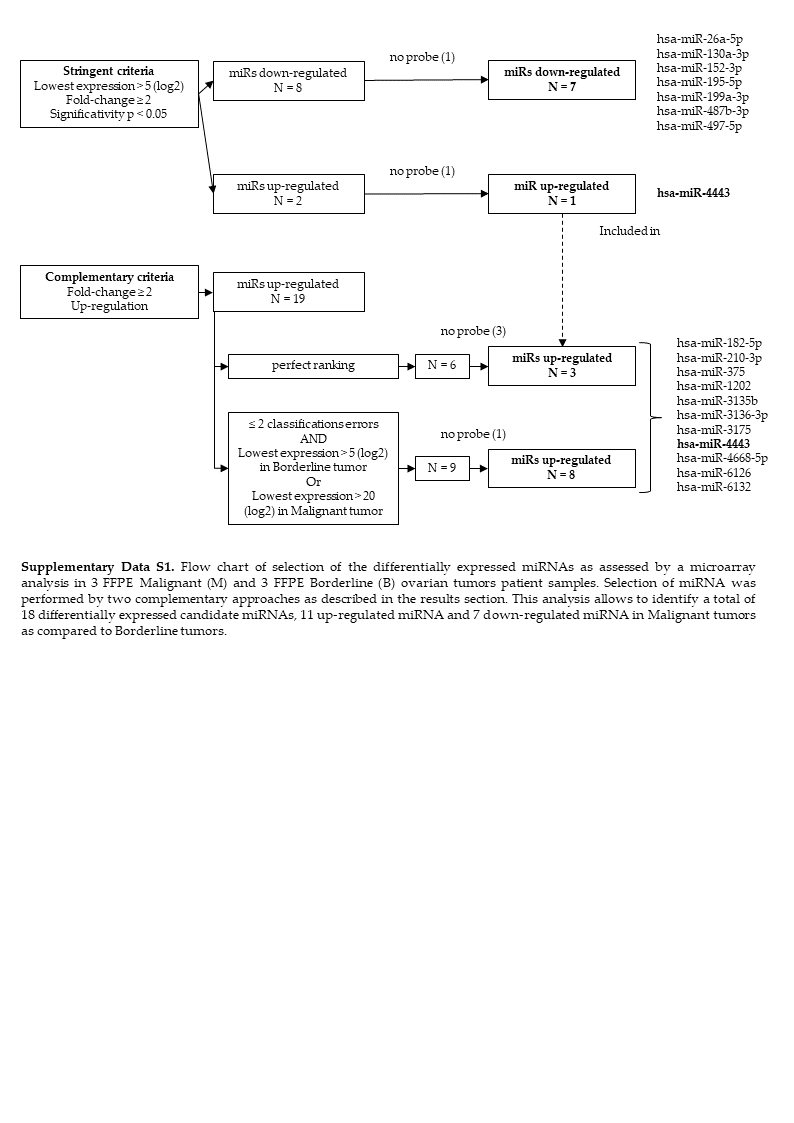

Supplement: Supplementary file 1 [file ijms-24-16016-s001.zip › Diapositive1.PNG]

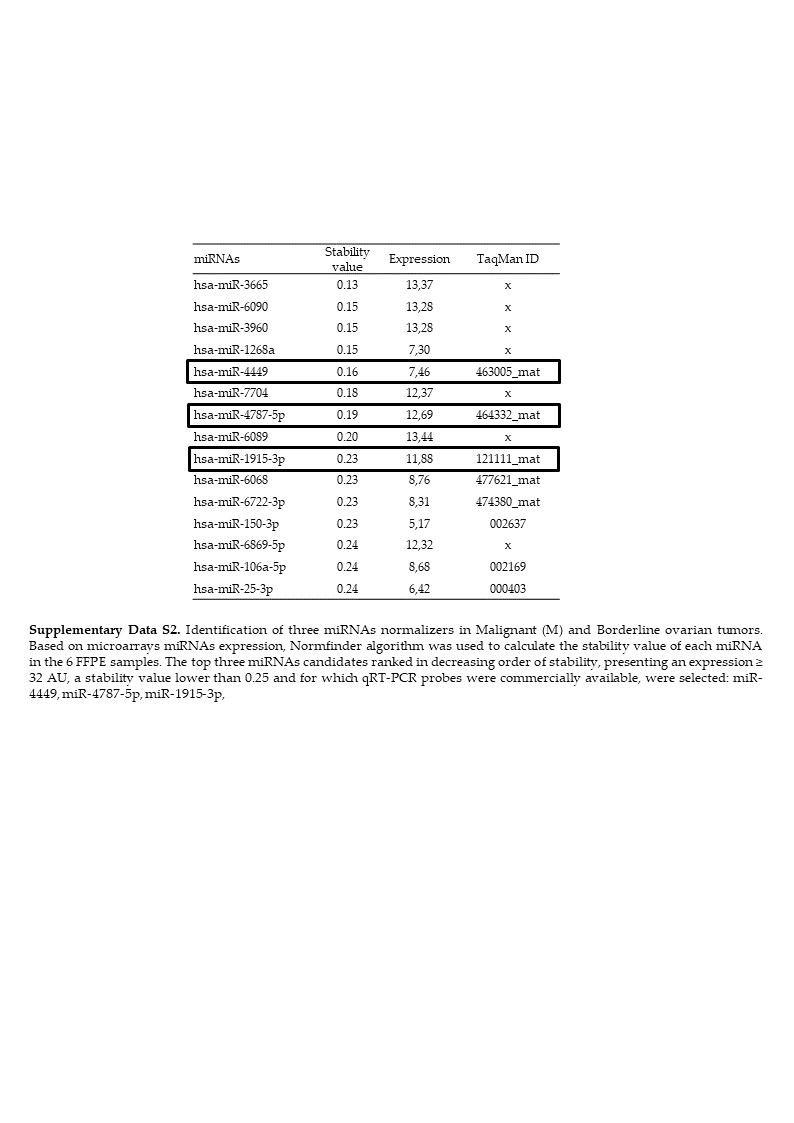

Supplement: Supplementary file 1 [file ijms-24-16016-s001.zip › Diapositive2.PNG]

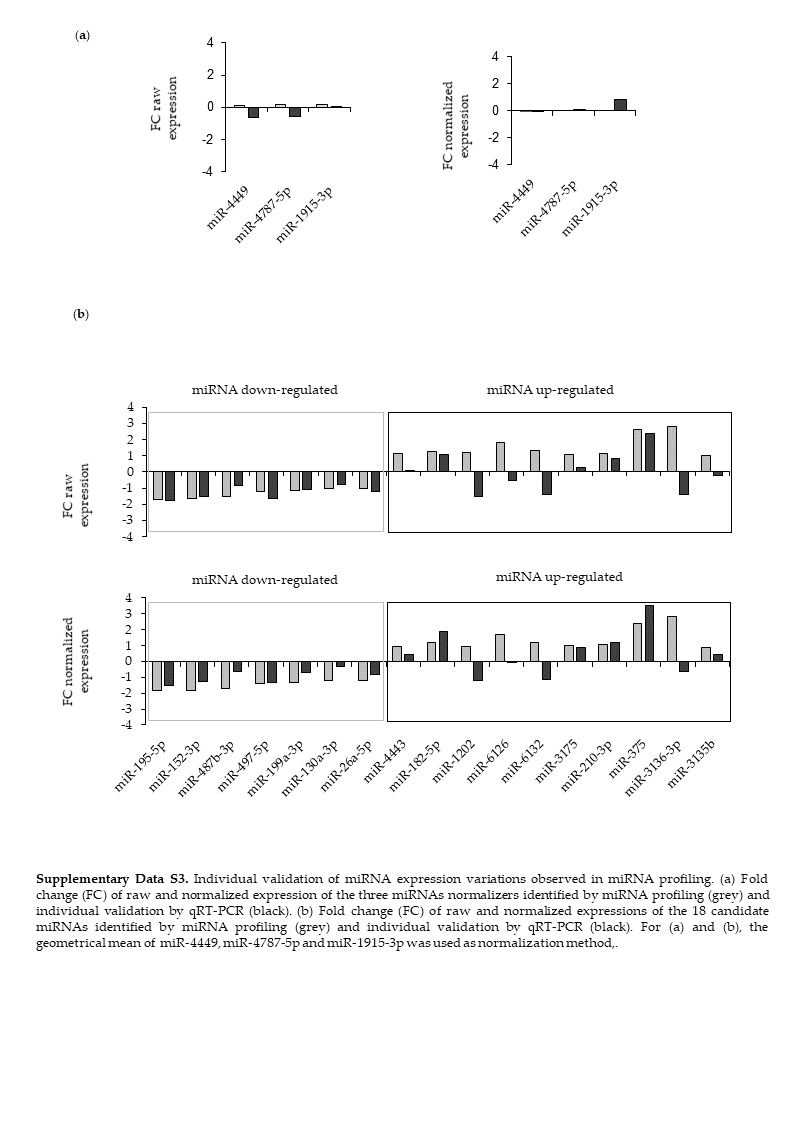

Supplement: Supplementary file 1 [file ijms-24-16016-s001.zip › Diapositive3.PNG]

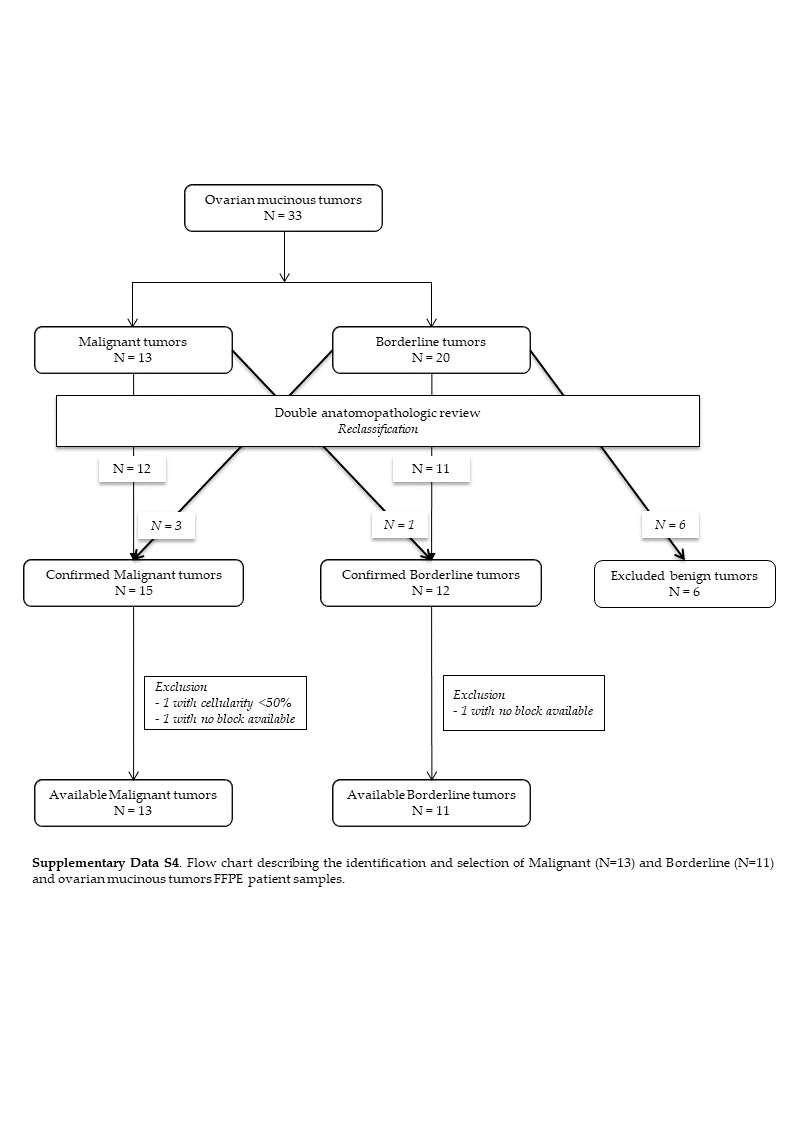

Supplement: Supplementary file 1 [file ijms-24-16016-s001.zip › Diapositive4.PNG]

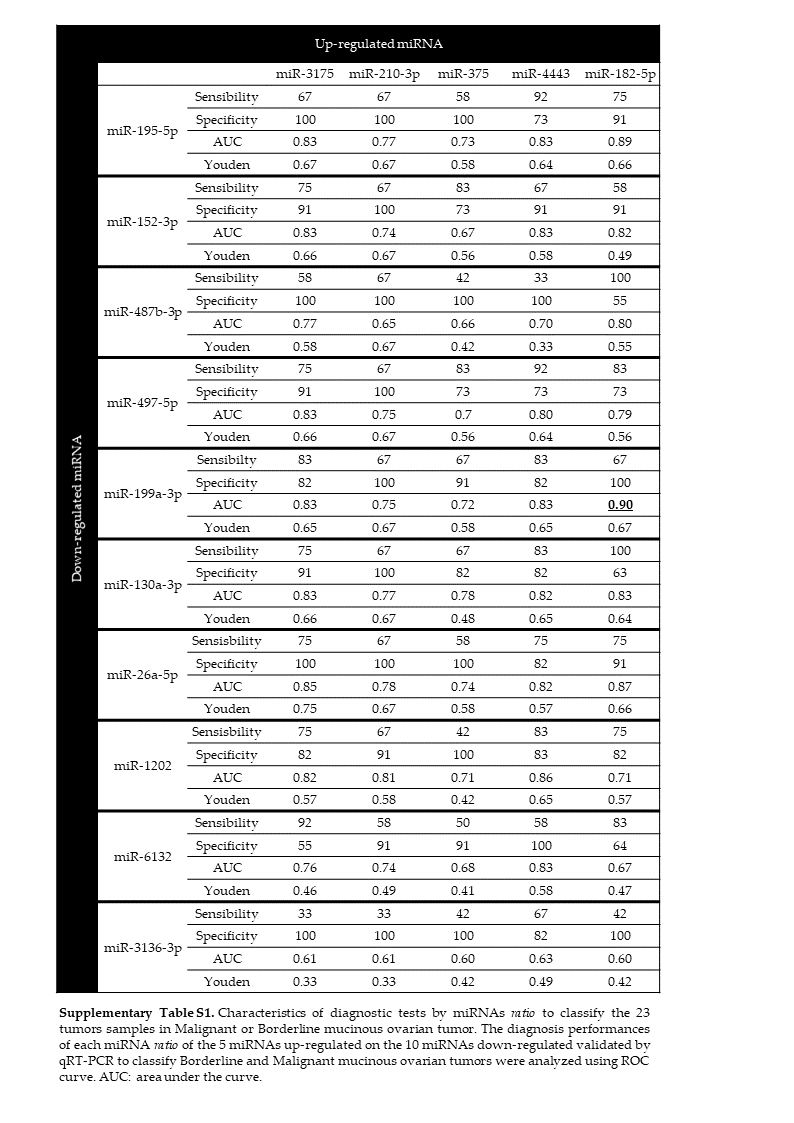

Supplement: Supplementary file 1 [file ijms-24-16016-s001.zip › Diapositive5.PNG]
